# A physical activity intervention to improve the quality of life of patients with a stoma: a feasibility study protocol

**DOI:** 10.1186/s40814-019-0461-2

**Published:** 2019-06-17

**Authors:** Gill Hubbard, Rebecca J. Beeken, Claire Taylor, Angus J. M. Watson, Julie Munro, William Goodman

**Affiliations:** 1 Department of Nursing and Midwifery, University of the Highlands and Islands, Centre for Health Science, Old Perth Road, Inverness, IV2 3JH Scotland, UK; 2 Leeds Institute of Health Sciences, University of Leeds, Worsley Building, Clarendon Way, Leeds, LS2 9NL England, UK; 3 St Mark’s Hospital, London North West University Healthcare NHS Trust, Harrow, Middlesex, HA1 3UJ England, UK; 4 Department of Surgery, Raigmore Hospital, NHS Highland, Old Perth Rd, Inverness, IV2 3UJ England, UK; 5 Research Department of Behavioural Science and Health, University College London, Gower Street, London, WC1E 6BT England, UK

**Keywords:** Ileostomy, Colostomy, Stoma, Bowel cancer, Inflammatory bowel disease, Physical activity, Quality of life

## Abstract

**Background:**

Physical activity (PA) is positively associated with quality of life. People with a stoma are less likely to engage in PA than those without a stoma.

**Methods:**

In this feasibility intervention study, we will perform the following: (1) Develop a PA intervention for people with a stoma. An Expert Working Group of behavioural scientists, exercise scientists, clinicians and a Patient Advisory Group of people with a bowel stoma will meet with the research team to inform the development of a PA intervention for people with a stoma. A manual of the intervention will be the main output. (2) Explore PA instructors’ experiences of delivering the PA intervention. PA instructors will record on paper the number of PA consultations with each patient and a researcher will interview the PA instructors about their experiences of delivering the intervention. (3) Assess the level of patient (bowel cancer or inflammatory bowel disease (IBD) patients with a stoma between 6 weeks and 24 months post-surgery) engagement with the PA intervention and their views on intervention acceptability and usefulness. Patients will keep a PA diary to record daily pedometer recorded step count and type and duration of activities. A researcher will interview patients about their experiences of the PA intervention. (4) Assess screening, eligibility, consent, data completion, loss to follow up, and missing data rates, representativeness of participants and potential treatment effects. A researcher will record on paper all study procedure parameters. Quality of life (stoma-quality of life; Functional Assessment of Cancer Therapy, Short IBD questionnaire), fatigue (FACIT fatigue scale) and PA (accelerometer) will be measured pre- and post-intervention in patients. For IBD patients only, blood will be taken to measure systemic inflammation.

**Discussion:**

We hypothesise that a PA intervention will be an effective means of improving the quality of life of people with a stoma. Before embarking on a full randomised controlled trial to test this hypothesis, a PA intervention needs to be developed and a feasibility study of the proposed PA intervention conducted.

**Trial registration:**

ISRCTN58613962, Protocol version: 0.1. 14 September 2017.

## Background

This article presents a research protocol for a feasibility study of a physical activity (PA) intervention to improve the quality of life in patients with a bowel stoma. The Standard Protocol Items: Recommendations for Interventional Trials (SPIRIT) checklist for reporting intervention trial protocols has been used for guidance [1].

A stoma is an artificial opening on the surface of the abdomen that has been surgically created in order to divert the flow of faeces or urine [2]. The two types of eliminating bowel stomas are colostomy and ileostomy, which can be temporary or permanent [2]. In Europe, approximately 700,000 people are living with a stoma, and in the USA, more than 1 million people have a stoma [3]. It is estimated that there are 100,000 people with stomas in the UK [4]. There are a number of conditions that may necessitate the formation of a bowel stoma including bowel cancer and inflammatory bowel disease (IBD) [2]. Recent systematic reviews suggest that a stoma has a negative impact on quality of life [5–7]. Research about lived experiences and psychosocial health following stoma formation highlights three key themes: psychosocial impact around feeling of loss of control of body function, physical aspects that affect psychological function and quality of life and the process of acceptance, adaptation and adjustment [8]. A systematic review of 11 qualitative studies about people’s experiences of bodily change following stoma formation suggests that people experience profound disruptions in how their body looks, functions, sounds, smells and feels [9]. Interventions are therefore needed that have the potential to improve the quality of life for this group of patients.

PA has been identified by patients with a stoma as a research priority in relation to their quality of life [10]. A recent systematic review found that PA was positively associated with quality of life in long-term (≥ 5 years post diagnosis) bowel cancer survivors [11]. Additionally, emerging evidence suggests an association between PA and quality of life in people with IBD [12]. Yet, in bowel cancer survivors, those with a stoma are less likely to engage in PA than those without a stoma (odds ratio (OR) = 1.51, 95% confidence interval (CI) = 1.12–2.04) [13]. Furthermore, two recent surveys found that people with a stoma report reductions in PA following stoma formation [14, 15]. To address low levels of PA, there is a need to develop PA interventions specifically for people with a stoma.

## Methods

### Aim

We hypothesise that a PA intervention will be an effective means of improving the quality of life of people with a stoma. Before embarking on a full randomised controlled trial to test this hypothesis, a PA intervention needs to be developed and a feasibility study of the proposed PA intervention conducted. The main focus of this study is feasibility and acceptability of intervention implementation and study procedures for recruitment and measures of treatment effects. The objectives of this feasibility study, therefore, are as follows: (1) to develop a PA intervention for people with a stoma; (2) to explore PA instructors’ experiences of delivering the PA intervention; (3) to assess the level of patient engagement with the PA intervention and their views on intervention acceptability and usefulness; and (4) to assess screening, eligibility, consent, data completion, loss to follow up, missing data rates, representativeness of participants and potential treatment effects.

### Design

The research will draw on Stage I Development and Stage II Feasibility and Piloting of the Medical Research Council framework for the development of complex interventions [16]. Stage 1 involves intervention development. In Stage 2, the feasibility and acceptability of the intervention and trial procedures will be assessed using a quasi-experimental design, which will include measures of potential treatment effects being taken pre- and post-intervention.

### Stage 1: Intervention development

The research team already have an idea of what the intervention will look like, which is based on their previous work with cancer patients, including people with bowel cancer [17, 18]. Nonetheless, an Expert Working Group of behavioural scientists, exercise scientists, clinicians and a Patient Advisory Group of people with a bowel stoma will meet with the research team to inform the development of a PA intervention for people with a stoma. A manual of the intervention will be the main output from Stage 1.

The plan is to develop an intervention so that people with a bowel stoma are referred to a PA instructor, trained by the research team, to prescribe and support people with stoma engage in PA. Participants will undergo a physical function and fitness test (6-min walk [19], 30-s sit-to-stand test and chair sit-and-reach and arm-curl test [20]), be offered weekly PA consultations for 12 weeks and receive a pedometer to monitor their weekly PA in a diary designed for the purposes of the study. The PA consultations will be individual, face-to-face or by video conference once a week for 12 weeks. To promote and sustain behaviour change, consultations will incorporate core behaviour change techniques (e.g. goal-setting) recommended by the National Institute for Health and Care Excellence [21].

### Stage 2: Feasibility study

#### Setting

Participants will be recruited from three National Health Service Trusts/Boards: NHS Highland (Scotland); London North West Healthcare Trust; University College London Hospitals NHS Foundation Trust.

#### Eligibility criteria

##### Inclusion

- Diagnosed with Stage I–IV bowel cancer OR diagnosed with IBD;
- > 6 weeks and < 24 months since stoma formation (permanent or temporary) surgery (laparoscopic or open surgical procedure); and
- Willing and able to provide written informed consent.

##### Exclusion

- Emergency surgery for stoma formation;
- Clinician recommends that the patient should not engage in any type of PA; and
- Ongoing adjuvant therapy (chemotherapy or radiotherapy).

#### Main outcomes of the feasibility study

The main focus of this study is feasibility and acceptability of intervention implementation and study procedures for recruitment and measurement of treatment outcomes.

##### Assessing feasibility and acceptability of intervention implementation

The intervention will involve participants having 12 consultations with a PA instructor. Intervention fidelity will be measured by the number of consultations completed. PA instructors will record for each participant the number of PA consultations, duration and type (e.g. face-to-face, video call, telephone call). The PA instructor will prescribe three types of physical activity during each PA consultation for each week: physical fitness/aerobic activities (e.g. walking, cycling, swimming), muscular strengthening activities and mobility and movement activities. The PA instructor will recommend level of exertion for each type of activity using the Borg RPE Scale [22].

Intervention adherence will be measured as the completion rate of the prescribed PA. The participant will write down the three types of prescribed weekly activities in a PA diary that they will complete for the 12-week duration. Participants will record for each day their daily step count from the pedometer. They will record how much of the prescribed activity they managed that week using a continuous rating scale: all of it (100%), most of it (75%), some of it (25%), and none of it (0%). Hence, intervention adherence will be measured by the average completion rate of the three different types of prescribed activities. They will describe any additional physical activities they did during the week, how they felt doing the prescribed activities (e.g. challenges) and if they had any issues with their stoma.

The acceptability of the intervention will be explored through semi-structured face-to-face or telephone interviews (depending on participant preference) with participants and PA instructors. A semi-structured interview is chosen as it allows flexibility with sequencing of questions and for following up on any topics that arise naturally through discussion [23]. These interviews will last approximately 30 min and cover participants’ and PA instructors’ opinions on perceived stoma-related barriers to PA, content of the PA intervention and its perceived relevance and usefulness in addressing stoma-related barriers to being physically active.

##### Assessing study procedures

Screening, eligibility, consent and data completion and loss to follow up rates and reasons for excluding patients will be presented as percentages. Recruitment rates will be measured as rate of invited participants who are eligible and consenting. Clinical information including diagnosis (e.g. cancer or IBD), surgery (e.g. open or laparoscopy), date of stoma surgery, disease staging, stoma type (e.g. permanent or temporary, ileostomy or colostomy) and demographic information (e.g. age, sex) will be collected to assess representativeness of participants. Suitability of measurement procedures will be evaluated based on completion rates and rates of missing data.

#### Other outcomes of the feasibility study

We will assess the feasibility and acceptability of the proposed outcomes for a future randomised controlled trial at baseline and follow-up. We will also estimate potential treatment effects.

##### Quality of life

Stoma-related quality of life (QoL) will be measured using Stoma-QoL [24]. Nineteen items are scored in relation to work/social function, sexuality/body image, stoma function, financial concerns and skin irritation. Overall satisfaction with life is scored from 0 to 100, with a higher score indicating better QoL. To our knowledge, no recommended minimal important differences (MIDs) have been published for this instrument.

Bowel cancer-related QoL will be measured using the Functional Assessment of Cancer Therapy (FACT-C) [25]. The instrument includes general and colorectal cancer-specific subscales. Thirty-seven items are scored in relation to physical, social, emotional and functional well-being, and a specific section for colorectal cancer, and stoma QoL. A higher score indicates better QoL. The recommended MIDs are 2–3 points for the colorectal cancer subscale and 5–8 points for the FACT-C total score [26].

IBD-related QoL will be measured using the Short Inflammatory Bowel Disease Questionnaire (SIBDQ) [27]. The instrument measures physical, social and emotional status, with a higher score indicating better QoL. An evaluation of the Patient-Reported Outcomes Measurement Information System in a large cohort of patients with IBDs recommended MIDs in the range of 2–6 points [28].

##### Fatigue

Fatigue will be measured using the FACIT Fatigue Scale, which is a short, 13-item questionnaire that measures an individual’s level of fatigue during their usual daily activities over the past week [29]. The score range is 0–52, with a score of < 30 indicating fatigue. The recommended MIDs are 3 points [30].

##### Physical activity

The amount of PA will be objectively measured using the Actigraph GT3X+ accelerometer (Actigraph LLC, Pensacola, FL, USA) [31]. It will be worn around the wrist and measures activity counts, steps, inclinometers and light and moderate to very vigorous PA. Accelerometers record movement in such a way that it can be translated into a number of different outputs, for example, total step count, bouts of PA at specified intensities or energy expenditure. Accelerometer devices will be initialised by a researcher as follows: (1) Device recording of PA will be set for 7 days, with the intention to gain at least 4 usable days of data for each participant (4 days is standard practice). (2) The date and time when the participant is scheduled to wear the device will be set. The sample rate will be set to 30 Hz. (4) The unique participant ID will be added to the specific device. Once the device is returned by a participant, Actigraph software will be used to download data as follows: (1) the unit of measurement will be set at 10-s epochs; (2) the ‘# of axis’ setting will be set to 3; and ‘steps’, ‘lux’, ‘inclinometer’ and ‘low frequency extension’ will all be selected. Actigraph software wear-time validation will be set to meet the following criteria: (1) minimum number of valid days required = 4, (2) non-wear-time will be set at > 60 min of consecutive zeros and (3) minimum number of wear hours per day required will be set at > 10 h (600 min). Commonly accepted cut-off points for adults will be used to differentiate PA intensity using Freedson et al. [31] adult cut-off criteria: sedentary < 100 counts per minute, light 100–1951 counts per minute, moderate 1952–5724 counts per minute and vigorous > 5725 counts per minute. In addition, a sedentary bout will be set at 10 min.

##### Inflammation

Systemic inflammation is a marker of IBD disease activity. Hence, only a sub-group of participants (i.e. those with IBD) will have their blood taken. Inflammation will be measured by C-reactive protein, white cell count, neutrophil count, platelet count and albumin. As markers of inflammation, these laboratory results will indicate any changes in inflammatory response in the participant.

#### Participant timeline

**Fig. 1:**
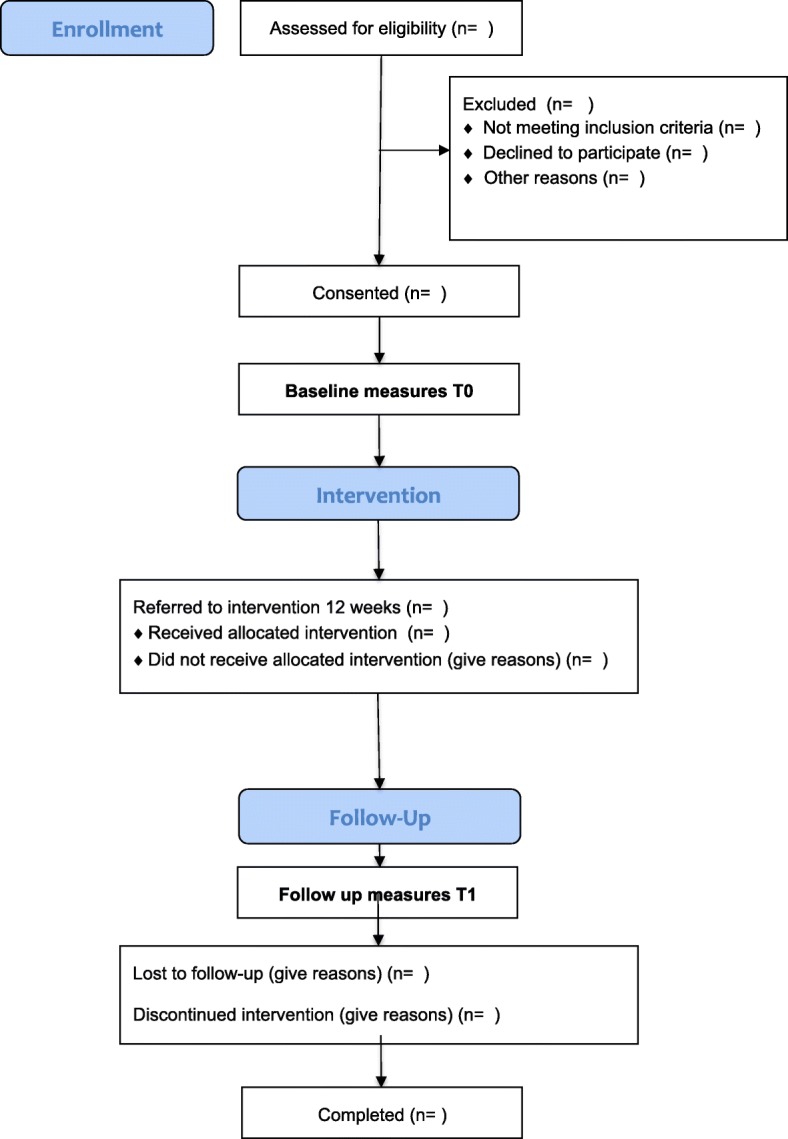
Participant Flowchart

**Fig. 2:**
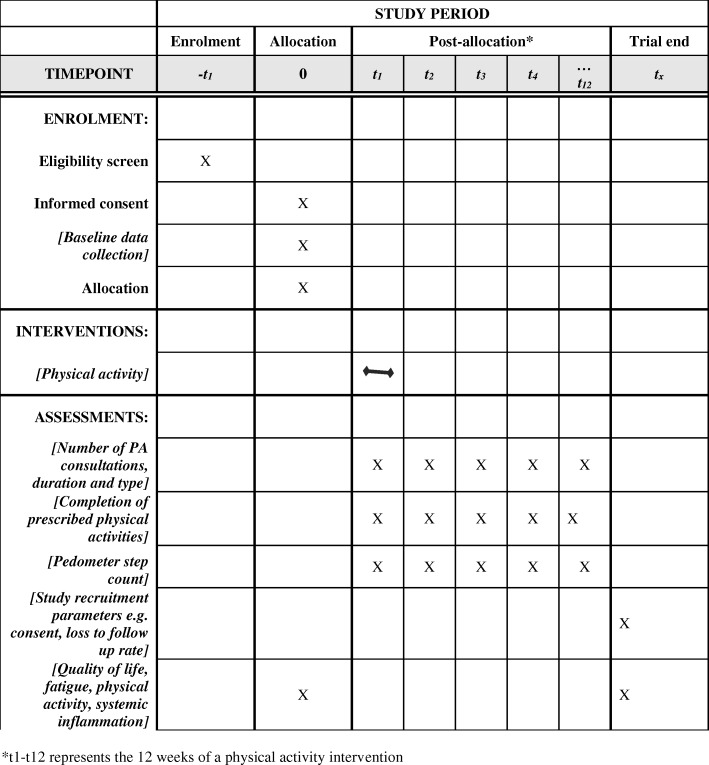
SPIRIT figure of enrolments, interventions and assessments. Asterisk denotes that t1–t12 represents the 12 weeks of a physical activity intervention

Figure 1 illustrates the process of enrolling participants in the study and timing of intervention and measurements. Figure 2 displays the SPIRIT figure of enrolments, interventions and assessments.

#### Sample size

It is inappropriate to base feasibility study sample sizes on measures of intervention effect, which is the purpose of the full-scale trial. Although how many participants are required in a feasibility study remains equivocal, suggestions range from 10 to 75 [32]. We aim to recruit 30 participants across three sites which should provide a sample of participants with different demographic and clinical characteristics that will improve our confidence in the conclusions we draw from this feasibility study.

#### Recruitment

Three recruitment methods will be used and the number of patients recruited by each method will be compared:

##### Prospective

A stoma/colorectal nurse specialist will discuss the study with eligible patients and, if they are interested, will pass their contact details on to a member of the research team. Verbal consent will be obtained. A researcher will then contact the patient to organise a face-to-face meeting where they will explain the study in further detail and take informed consent from the participant.

##### Retrospective

A list of potentially eligible patients will be put together by the clinical team. This list will be anonymised by removing name and contact details so that a member of the research team can re-assess patients for eligibility. The clinical team will send a letter of invitation to those that are eligible. Those that are interested will get in touch with the research team so that eligibility can be confirmed and to explain the study in more detail and take informed consent.

##### Social media

Recruitment via social media will also be used to maximise recruitment numbers. An advertisement about the study will be disseminated by members of the Patient Advisory Group and by relevant stoma charities on both Facebook and Twitter. Contact details of the research team will be provided, along with brief eligibility criteria. This will allow anyone who is interested in taking part to see if they will be eligible and to contact the research team directly.

#### Data collection

Data to evaluate the main outcomes of the feasibility study will be collected as follows: to collect data to assess intervention implementation, PA instructors will keep a paper record of each PA consultation with each participant; to collect data to assess intervention adherence, participants will complete a PA diary; and to collect data to assess study procedures, researchers in the three sites will keep records of recruitment parameters (e.g. number of participants screened, consenting) and completion of baseline and follow up measures.

Data to evaluate the other outcomes of the feasibility study will be collected as follows: proposed outcome measures for a future randomised controlled trial will be collected twice immediately before and immediately after the 12 week PA intervention. Each participant will meet with a researcher face to face to complete questionnaires (QoL and fatigue measures) hosted by Bristol Online Survey, which is an online service that allows researchers to develop, deploy and analyse an online survey. Participants with IBD only will have their blood taken by a nurse and the researcher will send the sample to the hospital laboratory for testing. Participants will be given an accelerometer that will be worn during waking hours for 7 consecutive days for 1 week. At the end of the 7-day period, participants will return the device to the research team by post.

#### Data analysis

Quantitative data analysis will be conducted in three steps. First, descriptive statistics will be used to describe demographic and clinical characteristics of the studied population stratified by eligible consenting and non-consenting participants, and screening, eligibility, consent and data completion rates and reasons for excluding patients reported as percentages. Second, intervention fidelity and intervention adherence continuous data (e.g. number of PA consultations, pedometer step count) will be presented as means and standard deviation and categorical data (type of consultation) as percentages. Third, assuming continuous data outcomes, differences pre- and post-intervention will be presented by the mean difference with the associated 95% confidence interval following *t* test analysis. This is because the focus of the results from this feasibility study will be on the estimates of treatment effects rather than statistical significant and hypothesis testing, which is the purpose of a full trial. Qualitative thematic analyses of audio-recorded interviews will be conducted using the Framework approach which is a rigorous method providing a structure within which qualitative data are organised and coded and themes identified [33].

#### Ethical considerations

The research study has been approved by NHS North East Scotland (REC reference 17/NS/0065); IRAS project ID: 219548).

##### Consent

The ethical principles of ensuring freely given fully informed consent and the right to withdraw from research participation will apply. The right to anonymity when reporting findings will be emphasised.

##### Confidentiality

All participants will be informed that all of the information that they provide to the research team will remain confidential and will only be accessible to members of that team. Only personal information that is deemed vital for running this study will be obtained. Participants will be given a unique study identifier so that their name will be filtered out of any quantitative and qualitative datasets used for analysis.

##### Participant risk

The Association of Stoma Care Nurses (ASCN) UK recommends core muscle exercises to strengthen the abdominis in order to prevent parastomal hernia formation. However, ASCN also advise against lifting heavy objects since this may cause a parastomal hernia [34]. The American College of Sports Medicine roundtable on exercise guidelines for cancer survivors recommends for people who have a stoma that (a) physician-permission is obtained prior to participation in contact sports, (b) special consideration be put in place for swimming activities, (c) resistance programmes should begin with low resistance and slow progression and (d) flexibility programmes should pay particular attention to maintaining correct breathing patterns in order to avoid excessive intra-abdominal pressure [35]. We will ensure that all these recommendations are covered in the training programme for the instructors delivering the PA intervention in this feasibility study.

##### Safety reporting

The adverse event reporting procedures will follow those of NHS guidelines for research trials. Reporting of adverse effects will be included in participant diaries, and all participants will be advised and encouraged to report concerns to the research team, their stoma nurse and their PA instructor. All serious adverse events (SAE) and adverse events (AE) will be recorded. Any SAE or AE will be reported, regardless of considered link with intervention participation.

#### Data management

The study includes three paper report forms: researchers in each site will complete a ‘recruitment form’ indicating if a patient is eligible to participate and screened patient clinical information (e.g. diagnosis); PA instructors will complete a ‘consultation form’ indicating duration and type of consultation; participants will complete a PA diary (e.g., prescribed activities). Data on paper report forms will be manually entered by a researcher into customised password encrypted spreadsheets in Microsoft Excel. A researcher will export data entered into Bristol Online Survey to the Statistical Package for the Social Sciences v19.0 for the purposes of analysis. All electronic data will be retained on a university password-protected server, and all paper records will be retained in a secure storage facility on university premises for a minimum of 10 years.

## Discussion and dissemination

The findings from this feasibility study will be shared with interested parties and audiences on a national and international level. The intention of this feasibility study is to inform a full randomised control trial, and any outcomes and findings from this preliminary work will be disseminated on that basis.

Evidence suggests that feasibility studies do not guarantee success for the future trial and this is often due to poor recruitment [36]. There are a range of contextual factors influencing the success of a trial (e.g. delays in study starting due to research governance procedures, poor recruitment due to poor buy-in from the clinical team) and wide variation between sites, which is why in the future full randomised controlled trial there will be an internal pilot in all sites [36].

We will present the study findings to the Big Bowel Event which is organised by a UK research charity—Bowel and Cancer Research. Participants at the Big Bowel Event include patients with a stoma, clinicians and researchers. The audience will help the research team improve the intervention and study procedures in preparation for a randomised controlled trial. A limitation of this feasibility study is that we will not gather data about the acceptability of randomisation. We will, however, seek advice about what is the most acceptable comparator arm in a future full trial for example, wait-list control or usual care. A further limitation is that the feasibility study will not provide indication of the sample size needed for a future trial. We will seek advice at the Big Bowel Event regarding what would be a meaningful difference in outcomes between patients receiving a PA intervention and those who do not from a patient perspective and use their recommendation to estimate the sample size needed for a future trial. We will inform participants of the key findings if they agree to receive this information. We will share information at relevant conferences, and events of interest to the population involved. We will also provide a lay summary of the trial findings for public dissemination through charities, support groups and other interested parties.

Study recruitment began in December 2017 and is currently ongoing.

To the best of our knowledge, this is the first PA intervention study for patients with a stoma. There remains a limited evidence base about PA in the stoma population. Cross-sectional cohort studies highlight a trend toward inactivity after stoma formation surgery and a fear of exercise in general [14, 15]. However, there have been no PA intervention studies to date. This feasibility study represents crucial preliminary work leading to a multi-site, full randomised controlled trial.

## Data Availability

The datasets used and/or analysed during the current study are available from the corresponding author on reasonable request.

## Abbreviations

AE: Adverse event
CI: Confidence interval
FACIT: Functional Assessment of Chronic Illness Therapy
FACT-C: Functional Assessment of Cancer Therapy-Colorectal
IBD: Inflammatory bowel disease
MIDs: Minimal important differences
OR: Odds ratio
PA: Physical activity
QoL: Quality of life
SEA: Serious adverse event
SIBDQ: Short Inflammatory Bowel Disease Questionnaire
SPIRIT: Standard Protocol Items: Recommendations for Interventional Trials
